# Factors influencing the adoption of secure software engineering practices in pre-adoption and post-adoption phases

**DOI:** 10.1038/s41598-025-27024-7

**Published:** 2025-11-28

**Authors:** Anže Mihelič, Tomaž Hovelja, Simon Vrhovec

**Affiliations:** 1 Faculty of Criminal Justice and Security, University of Maribor, Kotnikova 8, 1000 Ljubljana, Slovenia; 2 Faculty of Computer and Information Science, University of Ljubljana, Večna pot 113, 1000 Ljubljana, Slovenia

**Keywords:** Software security, Software development, Secure software engineering, SSE, Theory of planned behavior, Technology acceptance, Information technology, Software

## Abstract

Secure software engineering (SSE) is inevitably linked to DevSecOps which aims to embed security at every phase of the software development life cycle. The adoption of SSE practices is however poorly understood, especially in various adoption phases. This study aims to address this gap by exploring what motivates individuals to adopt SSE practices, and investigating how this differs between the pre-adoption and the post-adoption phase. A survey of people working in software development (N=463) recruited through the Prolific platform was conducted to investigate the determinants of behavioral intention to practice SSE. The measurement instrument was validated with a confirmatory factor analysis. Covariance-based structural equation modeling (CB-SEM) was used to determine associations between constructs based on the theory of planned behavior (TPB), technology acceptance model (TAM), and awareness of security risks to developed software and the newest SSE practices. The results indicate that TPB, TAM and awareness explain adoption of SSE practices well. They also indicate major differences between the pre-adoption and post-adoption phases as none of the meaningful associations overlapped between the phases. In the pre-adoption phase, associations of behavioral intention with subjective norm and both awareness constructs have the meaningful effect sizes. Additionally, a non-significant association with perceived usefulness has a small effect size. In the post-adoption phase, behavioral intention was associated with attitude toward SSE and ease of use. Key implications of this study stem from the detected differences between adoption phases which should be considered both in future studies on this topic, and in practical settings in which SSE and thus DevSecOps is being implemented.

## Introduction

Secure software engineering (SSE) is a specialized area in software development that emphasizes integrating security measures into the software development life cycle. It is inevitably linked to DevSecOps which aims to embed security at every phase of the software development life cycle^1–4^. It introduces security early in the development process, enabling teams to identify and mitigate security risks sooner. This approach promotes collaboration across development, operations, and security teams as ensuring security becomes a shared responsibility. Continuous security checks may be embedded in every stage, from coding to deployment, helping tackle emerging threats and proactively maintaining compliance with industry standards. SSE is thus essential for mitigating risks and vulnerabilities, and is becoming increasingly important as software plays a critical role in various aspects of life and business as also outlined by international standards^5^. With the escalation of digital technology reliance, ensuring robust software against security threats – both intentional and unintentional – is vital, highlighting the indispensable role of SSE in modern software development^6,7^.

The effectiveness of SSE practices significantly depends on individual developers’ willingness and ability to adopt these practices. Understanding the factors associated with individual developers’ adoption of SSE is thus essential for creating a security-conscious culture within software development teams^8^. This understanding is vital for developing strategies and tools that support SSE, enhancing training programs, and ultimately leads to coding practices that are more secure. Therefore, the exploration of individual motivators and barriers in SSE adoption remains a key area of focus in software engineering research^9,10^.

Few studies explored SSE adoption, and each of them did it from a different perspective. One of the first studies on this topic employed the theory of planned behavior (TPB) and its predecessors to study the determinants of behavioral intention to practice SSE^11^. It indicates only partial support for TPB in this context as only attitude toward behavior and subjective norm were associated with behavioral intention while other factors, such as self-efficacy, were not^11^. Several later studies leaned on key adoption theories^12^. First, a study employing the diffusion of innovation (DOI) theory to explain adoption of SSE practices indicated associations between behavioral intent to adopt SSE methods and relative advantage, compatibility, and trialability^13^. Next, a study leaning on the technology acceptance model (TAM) found that behavioral intention to adopt secure coding methodologies was directly associated with perceived usefulness and only indirectly with ease of use^14^. Finally, a study proposed to apply the Unified Theory of Acceptance and Use of Technology (UTAUT) for explaining the adoption of SSE practices^15^ albeit we found no such empirical studies in the literature. Additionally, we found a study building on the technology-organization-environment (TOE) framework that investigated the roles of organizational (e.g., organizational support, organizational justice), technical (e.g., absorption capacity) and individual characteristics (e.g., interest and fun, social needs) in adoption of SSE methods^16^. To summarize, the presented studies do not combine different theories when studying adoption of SEE practices. They also do not differentiate nor investigate the differences between phases of the adoption process.

In this study, we aim to address the presented gaps in our understanding of SSE adoption. This study makes two key contributions. First, this is one of the first studies to study adoption determinants from competing theories (i.e., TPB and TAM^17^) therefore contributing to the literature on adoption of SSE practices. Second, it investigates differences between adoption determinants in the pre- and post-adoption phases. This is a step in the opposite direction of the classical adoption theory which aims to unify adoption phases into a single framework^18^ and therefore contributes to the adoption theory both in the context of SEE practices as well as in general.

## Theoretical background

Any technology an enterprise aims to implement into work practice should firstly be adopted by its users. As (secure) software development methods are considered a technology^19^, several authors investigated factors predicting acceptance of secure software development practices through technology adoption theories. One of the most prevalent theories explaining why some innovations get accepted faster while others remain nearly untouched is explained by Rogers’ diffusion of innovation theory (DOI). It explains how a particular innovation idea diffuses over time through a social system^20^. The principles of DOI theory were readily included in research in the software development research as the basis for understanding the implementation of particular methodology or developing new models (e.g.,^21–23^).

The second set of most significant theories used in research on adoption of software development methods among individuals are the technology acceptance model (TAM)^24^ and its derivatives (e.g., TAM2^25^)^22,26^. The original theory is based on three core constructs: 1) perceived usefulness of the technology, 2) perceived ease of its use, and 3) the intention to adopt the technology. Additionally, a target construct is the actual use of the technology. The succeeding TAM2 adds several more factors. The most significant of them (i.e., the one directly influencing the intention) is the subjective norm. Subjective norm refers to a belief of potential user that people who are important to them will approve and support the adoption of particular technology^27^. Apart from subjective norm, perceived usefulness is predicted by an image, job relevance, output quality, and result demonstrability, while subjective norm is moderated by voluntariness and experience^28^.

Venkateshs’ unified theory of acceptance and use of technology (namely, UTAUT and UTAUT2)^29,30^ and Ajzens’ theory of planned behavior (TPB)^31^ are the final established theories in literature on software development enterprises’ methodology adoption. Both are typical individual-centric theories for predicting technology-related behavior. TPB, which is a widely used theory in variety of topics, such as^32–35^, is based on three core constructs: 1) subjective norm, 2) attitude toward behavior, and 3) perceived behavioral control (a degree to which an individual believes performing particular behavior is easy or difficult^36^). These predict behavioral intention, while the latter finally predicts an actual behavior. UTAUT approaches a similar problem slightly differently - with a more technology-focused approach. Behavioral (adoption) intention is predicted by four factors: 1) expectation of performance, 2) expectation of effort, 3) social influence, and 4) facilitating conditions^29^. UTAUT2 adds additional factors to the original UTAUT model (i.e., hedonic motivation, price value, and habit)^30^. While the UTAUT2 adds additional factors as direct predictors of intention, it drops voluntariness from moderating factors. Hence, it only keeps gender, age, and experience^30^. Both theories were used in the field of software development in previous research (e.g., TPB^26,37,38^, UTAUT(2)^15,39,40^, however, some of the factors proposed by UTAUT(2) may not seem entirely suitable for predicting adoption of SSE practices.

When it comes to understanding the factors driving the process of adoption of SSE practices, the process depends not only on technical aspects but also on fostering a culture of open communication, shared responsibility, respect, and trust (as in Dev(Sec)Ops)^26^. In secure software development and promoting a security-conscious culture, individual motivation and perception should be considered. The security culture, for example, in open-source software communities, is shaped by the concepts found in above-mentioned theories, the attitudes, behaviors, and skills of the members^38^. Furthermore, the adoption of secure software practices in companies also relies on individual motivation, backed by organizational support and the ability to learn and adapt^16^. In small and medium enterprises, factors like the perceived advantages and compatibility with existing practices might also be considered when deciding whether to adopt secure software practices^13^. Additionally, a supportive community, including social and organizational backing, is essential for encouraging developers to adopt secure coding practices^14^.

## Research model

Literature in the field of cybersecurity awareness suggests a potential association between developers’ understanding of security risks and their behavioral intentions toward secure software engineering (SSE). Information security research has shown that awareness of potential risks significantly influences users’ and developers’ attitudes and intentions toward protective behaviors^41,42^. In software development contexts, this includes recognizing threats such as injection attacks, misconfigurations, or data leakage. Studies have identified a gap in security-centered programming awareness among developers, which highlights a need for increased focus in this area^43–46^. Despite this, risk awareness remains a key precondition for secure behavior. Therefore, we hypothesize: 

### H1

Awareness of risks to developed software is positively associated with behavioral intention to practice SSE.

Further,^42^ explored the influence of threat awareness on adopting protective measures, suggesting a positive correlation between awareness and behavioral changes. However, this relationship is not always straightforward, as evidenced by the ”knowing-and-doing” gap in cybersecurity, where awareness does not consistently lead to secure behaviors^47^. Understanding the relationship between awareness and behavior suggests a complex association that needs more exploration in software development. Accordingly, we hypothesize: 

### H2

Awareness of newest SSE practices is positively associated with behavioral intention to practice SSE.

The TPB^31^ proposes predicting factors of individual behavior as attitude, subjective norm, and perceived behavioral control. Various studies have previously applied this theory to predict behavior in SSE^11,15,41,48^. Within the TPB framework, attitude refers to the degree to which a person evaluates desirable or unfavorable behavior, in this case, secure coding practices^48^. Research (e.g.,^11^) has shown that attitude is a strong predictor in TPB, particularly in the domain of SSE, where the tendency to adopt secure practices is (among others) influenced by one’s attitude toward these practices. Additionally, experiences with security issues tend to have a lasting impact on attitudes toward software security^43^. The latter study indicated that after encountering security issues, most participants acknowledged the importance of software security and implemented specific procedures to address it. This lasting effect of experience on attitude suggests that a positive attitude toward SSE significantly contributes to the intention to engage in secure coding practices. Based on this, we hypothesize: 

### H3

Attitude toward SSE is positively associated with behavioral intention to practice SSE.

Subjective norm, as described in the TPB and emphasized by numerous researchers, plays a critical role in shaping behavioral intentions^31^, including those related to secure software engineering (SSE). These norms represent the perceived social pressure exerted by influential groups or individuals, which can significantly influence a person’s opinions and attitudes^48,49^. In the context of SSE, the influence of subjective norms has been acknowledged in various behavioral prediction models^11,41,48^. These norms can be explained by the perception of what significant others think about performing or not performing a behavior^41^. However, it’s important to note that subjective norm does not always significantly predict behavioral intention^42^, even though it was proven as a significant predictor of behavioral intention regarding SSE practice^11^. In work-related issues, especially in SSE, colleagues often serve as important referents, influencing individual behaviors through subjective norm^16^. Subjective norm is particularly relevant in the field of SSE because they cause collective expectations and pressures within the professional community^41^. In software development environments, the influence of peers, management, and the broader organizational culture can significantly shape a developer’s approach to secure coding. The adoption of SSE practices is not just a matter of individual skill or awareness but is also influenced by the perceived expectations and norms within the work environment. Based on this, we hypothesize: 

### H4

Subjective norm is positively associated with behavioral intention to practice SSE.

When it comes to technology adoption, one of the main theories used in the literature is the Technology Acceptance Model^24^ and its derivatives, which typically means including the potential ease of use of the technology and its perceived usefulness. In the field of software engineering, especially SSE, the concept of ease of use has not been frequently examined, in contrast to perceived behavioral control, which has been a more common focus^11,16,41,48^, even though literature suggests a strong association between the two constructs^42^, where it should be emphasized, that perceived behavioral control refers to the individual’s perception of the ease or difficulty using a technology^50^ when applying it to the context of adopting new technology. However, ease of use, known as a vital predicting factor in adopting new technologies across various fields^25^, including engineering^51^, together with its strong link to perceived behavioral control^42^, suggests its relevance in SSE. Thus, we build on the premise that if SSE practices and tools are perceived as easy to use, they are more likely to be adopted and integrated into regular development workflows. This is rooted in the assumption that ease of use makes new practices more approachable and plays a similar (even though not exactly the same) role as perceived behavioral control. Therefore, we hypothesize: 

### H5

Ease of use is positively associated with behavioral intention to practice SSE.

Together with ease of use, perceived usefulness, another fundamental construct of the TAM^24^, plays a vital role in predicting the behavioral intention in technology adoption. It is often considered a stronger predictor than ease of use^12^, and reflects the degree to which developers believe that employing specific security measures or tools will enhance their (task) performance. As such, it is a key driver for their adoption in the domain of security practices^11^. Additionally, the complexity of security tools, which negatively impacts their perceived usefulness, can discourage their adoption^23^. The importance of perceived usefulness in encouraging the adoption of security tools and practices is further suggested in studies on SSE^43,52^. However, the perceived usefulness extends beyond only the functionality of tools and also includes the broader benefits of the software development process itself^53^. In this context, the perceived usefulness of SSE, which contains both the direct benefits and the perceived advantages of the development process, we hypothesize that perceived usefulness is positively associated with the behavioral intention to adopt and adhere to secure coding practices: 

### H6

Perceived usefulness is positively associated with behavioral intention to practice SSE.

## Research methodology

### Research design

This study employed a cross-sectional research design to determine which factors are associated with adoption of SEE practices. A survey was conducted to investigate the determinants of behavioral intention to practice SSE.

### Ethical considerations

The study proposal was approved on 8 April 2022 by the Research Ethics Committee of the University of Maribor, Faculty of Criminal Justice and Security. All research was performed in accordance with relevant guidelines and regulations.

### Measures

Theoretical constructs were defined and operationalized as presented in Table 1. Questionnaire items were taken from previously validated research and adapted to the context of the study. All theoretical constructs were modeled as first-order reflective constructs. Items for *awareness of risks to developed software* and *awareness of newest SSE practices* were adapted from^41^. Next, items for *attitude toward SSE*, *subjective norm* and *perceived usefulness* were adapted from^11^. Items for *ease of use* were adapted from^24^. Finally, items for *behavioral intention* were adapted from^54^. Questionnaire items for *awareness of risks to developed software*, *subjective norm*, *ease of use* and *perceived usefulness* were measured by using a 5-point Likert scale from 1 *strongly disagree* to 5 *strongly agree*. Questionnaire items for *awareness of newest SSE practices*, *attitude toward SSE* and *behavioral intention* were measured by using a 7-point Likert scale from 1 *strongly disagree* to 7 *strongly agree*. The survey was distributed in English.

**Table 1:** Definitions of theoretical constructs.

Theoretical construct	Operational definition
Awareness of risks to developed software [AoRtDS]	An individual’s awareness of risks to developed software.
Awareness of newest SSE practices [AoNSSEP]	An individual’s awareness of newest SSE practices.
Attitude toward SSE [AtSSE]	An individual’s positive versus negative evaluations of practicing SSE.
Subjective norm [SN]	The perception of social approval from important others regarding practicing SSE.
Ease of use [EoU]	The perceived ease of practicing SSE.
Perceived usefulness [PU]	The perceived usefulness of practicing SSE.
Behavioral intention [BI]	The intention to practice SSE.

### Sample and data collection

The sample included 463 people working in software development (e.g., software developers, security experts, project managers). The survey was conducted between 11 and 12 April 2022. Respondents were recruited through the *Prolific* platform https://prolific.co/. They were presented with an informed consent statement on the first page of the online survey. By proceeding to the survey, they indicated their agreement through implied consent (i.e., by continuing with the survey, they gave their consent through conduct). Respondents received a compensation of GBP 0.43 for taking the survey. A total of 530 respondents took the survey. 30 respondents did not complete the questionnaire or withdrew from the study. 14 responses were excluded for failing an attention check. Next, we excluded 22 respondents with no software development experience. Responses were further checked for indications of respondent non-engagement (i.e., standard deviation equal to 0). After excluding a single response due to respondent non-engagement, we were left with 463 useful responses which were used in further analyses.

**Table 2:** Sample characteristics.

Characteristic		Frequency	Percent (%)
Gender	Female	112	24.2
	Male	351	75.8
Formal education	Finished high school or less	86	18.6
	Received Bachelor’s degree or equivalent	261	56.4
	Received Master’s degree or equivalent	108	23.3
	Received PhD degree or equivalent	8	1.7
Role in software development	Software developer	345	74.5
	Security expert	6	1.3
	Project manager	61	13.2
	Other	50	10.8
	N/A	1	0.2
Country of residence	Australia	2	0.4
	Austria	1	0.2
	Belgium	2	0.4
	Canada	19	4.1
	Chile	4	0.9
	Denmark	1	0.2
	Finland	1	0.2
	France	7	1.5
	Germany	15	3.2
	Greece	12	2.6
	Hungary	8	1.7
	Ireland	1	0.2
	Israel	6	1.3
	Italy	29	6.3
	Korea	1	0.2
	Latvia	1	0.2
	Mexico	24	5.2
	Netherlands	5	1.1
	New Zealand	1	0.2
	Poland	41	8.9
	Portugal	74	16.0
	Slovenia	3	0.6
	South Africa	43	9.3
	Spain	21	4.5
	Sweden	3	0.6
	United Kingdom	98	21.2
	United States	40	8.6

Table 2 shows the sample characteristics. Age of the respondents ranged from 19 to 64 years old ($M=30.5,SD=8.7,median=28$). Experience with software development ranged from 1 to 36 years ($M=5.6,SD=6.1,median=3$). Experience with SSE ranged from 0 to 27 years ($M=1.7,SD=3.2,median=1$). A significant share of respondents ($N_{inexp}=202$) had no prior experience with SSE. A number of respondents chose a role in software development that did not fit out predefined categories (i.e., *Other*). These included quality assurance specialists, software testers, AI developers, various leading roles beside project managers, such as team and development leads, software architects, etc. A low share of *security experts* could be attributed to the fact that few people primarily specialize in security and may consider themselves primarily as some other role in software development even though they have experience with SSE. This is in line with the widely adopted agile methods in which a certain role can be played by several people, and a single person can play several roles^55,56^. Therefore, we considered experience with SSE as the key indicator of adoption of SEE.

### Data analysis

Covariance-based structural equation modeling (CB-SEM) was employed to analyze the data since all measured theoretical constructs were reflective. CB-SEM enables the analysis of complex research models when these include latent variables (i.e., theoretical constructs) with multiple indicators which allow for indirect measurement through characteristics attributed to them. A key advantage of CB-SEM is that it integrates the measurements of latent variables and associations between them into a concurrent evaluation.

Data was analyzed with *R* version 4.3.2, with *lavaan* version 0.6-16 and *semTools* version 0.5-6 packages. Missing values (0.05 percent) were imputed with medians prior to the analysis. When there is less than 10 percent missing values, any imputation method can be applied^57^. We considered median imputation as adequate since the reduced variance of the distribution and depressed observed variables induced^57–59^ is insignificant considering the relatively few missing data. Model fit of all measurement and structural models was determined with fit indices $\chi ^2/df$, comparative fit index (CFI), Tucker-Lewis index (TLI), root mean square error of approximation (RMSEA), and standardized root mean square residual (SRMR). Standard thresholds were used to interpret how well the data fit the models ensuring that the results of CB-SEM analysis are meaningful.

To validate the survey instrument, a confirmatory factor analysis (CFA) was conducted. *Convergent validity* was determined by evaluating average variance extracted (AVE) and factor loadings of items on their corresponding latent variables. *Discriminant validity* was evaluated with a heterotrait-monotrait ratio of correlations (HTMT) analysis. *Reliability* was determined with Cronbach’s alpha (CA) and composite reliability (CR).

A structural model was developed to test the hypothesized associations. We calculated Cohen’s $f^2$ for measuring local effect size for each hypothesized association^60^. This calculation includes the calculation of $R^2$ change for each association. There are various ways to calculate $R^2$ change for associations in CB-SEM. We opted to calculate $R^2$ change by using the direct matrix approach suggested by^61^ as it enables calculation of $R^2$ changes for all associations without the need to alter the structural model. We used function *rsquareCalc* in the provided script to calculate $R^2$ changes^61^.

To gain more insights into whether there are differences due to SSE experience, we split our sample into two subsamples. The *pre-adoption* subsample included respondents without SSE experience ($N_{pre}=202$). The *post-adoption* subsample included respondents with at least one year of SSE experience ($N_{post}=261$). First, we aggregated individual item scores into construct item scores by calculating their means. We used independent samples *t* tests to compare means of measured constructs between the subsamples. Next, we tested the structural model on each subsample to investigate the differences between the two subsamples.

## Results

### Measurement model

A measurement model was developed to validate the survey instrument. First, we tested the full measurement model. However, an item for *awareness of risks to developed software* (i.e., AoRtDS3) and *perceived usefulness* (i.e., PU3) had poor factor loadings and were thus excluded from the measurement model. The fit of the final measurement model presented in Table 3 indicates that the data fits it well.

**Table 3:** Fit indices of the measurement model.

Measure	Threshold	Estimate	Interpretation
$\chi ^2$		239.747	
*df*		131	
$\chi ^2 / df$	≤ 5	1.830	Excellent
CFI	≥ 0.90	0.984	Excellent
TLI	≥ 0.90	0.979	Excellent
RMSEA	≤ 0.08	0.042	Excellent
SRMR	≤ 0.08	0.034	Excellent

CFI – comparative fit index; TLI – Tucker-Lewis index; RMSEA – root mean square error of approximation; SRMR – standardized root mean square residual.

Table 4 presents CA, CR, AVE and HTMT analysis which are relevant for determining the validity and reliability of the survey instrument. First, CA ranged from 0.705 to 0.953 and CR from 0.710 to 0.954 exceeding the commonly accepted threshold 0.70. This demonstrates adequate reliability of all constructs. Next, AVE ranged from 0.553 to 0.874. Values above the 0.50 threshold are generally considered as acceptable therefore indicating adequate convergent validity. Additionally, all factor loadings (see Table 5) were above 0.50 showing further support for adequate convergent validity. Finally, HTMT ratios of correlations were all below the conservative 0.85 threshold thus indicating adequate discriminant validity of the survey instrument.

**Table 4:** Validity and reliability of the survey instrument: Cronbach’s alpha (CA), composite reliability (CR), average variance extracted (AVE), and heterotrait-monotrait ratio of correlations (HTMT) analysis.

Construct	CA	CR	AVE	1	2	3	4	5	6
1: AoRtDS	0.705	0.710	0.553						
2: AoNSSEP	0.953	0.954	0.874	0.428					
3: AtSSE	0.826	0.831	0.622	0.418	0.427				
4: SN	0.934	0.934	0.826	0.278	0.577	0.527			
5: EoU	0.894	0.895	0.740	0.378	0.596	0.416	0.461		
6: PU	0.716	0.717	0.558	0.381	0.144	0.657	0.219	0.213	
7: BI	0.929	0.930	0.817	0.417	0.614	0.669	0.632	0.542	0.407

AoRtDS – awareness of risks to developed software; AoNSSEP – awareness of newest SSE practices; AtSSE – attitude toward SSE; SN – subjective norm; EoU – ease of use; PU – perceived usefulness; BI – behavioral intention.

**Table 5:** Questionnaire items.

Construct	Loading	Item	Source
Awareness of risks to developed software	0.773	AoRtDS1. I am aware of potential security threats to developed software.	^41^
	0.707	AoRtDS2. I understand the risk of security exploits in developed software.	
	-	AoRtDS3. [excluded] I keep myself updated in terms of security possibilities for developed software.	
Awareness of newest SSE practices	0.948	AoNSSEP1. I am aware of the newest SSE practices.	^41^
	0.943	AoNSSEP2. I keep myself updated in terms of the newest SSE practices.	
	0.912	AoNSSEP3. I have sufficient knowledge about the newest SSE practices.	
Attitude toward SSE	0.855	AtSSE1. Practicing SSE is a good idea.	^11^
	0.753	AtSSE2. Practicing SSE is necessary.	
	0.777	AtSSE3. I like the idea of practicing SSE.	
Subjective norm	0.894	SN1. People who influence my behavior think that I should practice SSE.	^11^
	0.934	SN2. People who are important to me think that I should practice SSE.	
	0.896	SN3. People whose opinions I value prefer that I practice SSE.	
Ease of use	0.906	EoU1. Learning to practice SSE would be easy for me.	^24^
	0.866	EoU2. It would be easy for me to become skillful at practicing SSE.	
	0.810	EoU3. I would find practicing SSE easy.	
Perceived usefulness	0.768	PU1. Practicing SSE would make developed software able to better withstand attacks or misuse.	^11^
	0.726	PU2. Practicing SSE would enable security requirements to be better captured.	
	-	PU3. [excluded] Practicing SSE would reduce the costs of software maintenance (e.g., by avoiding retrofitting of security into developed software).	
Behavioral intention	0.913	BI1. I intend to practice SSE in the future.	^54^
	0.910	BI2. I will try to practice SSE.	
	0.891	BI3. I plan to practice SSE frequently.	

### Structural model

This subsection presents the results for the full sample. A structural model was developed to test the hypothesized direct effects. Model fit of the structural model is presented in Table 6, and indicates that the model fits the data well.

**Table 6:** Fit indices of the full sample structural model.

Measure	Threshold	Estimate	Interpretation
$\chi ^2$		252.069	
*df*		149	
$\chi ^2 / df$	$\le 5$	1.692	Excellent
CFI	$\ge 0.90$	0.984	Excellent
TLI	$\ge 0.90$	0.980	Excellent
RMSEA	$\le 0.08$	0.039	Excellent
SRMR	$\le 0.08$	0.034	Excellent

CFI – comparative fit index; TLI – Tucker-Lewis index; RMSEA – root mean square error of approximation; SRMR – standardized root mean square residual.

**Fig. 1:**
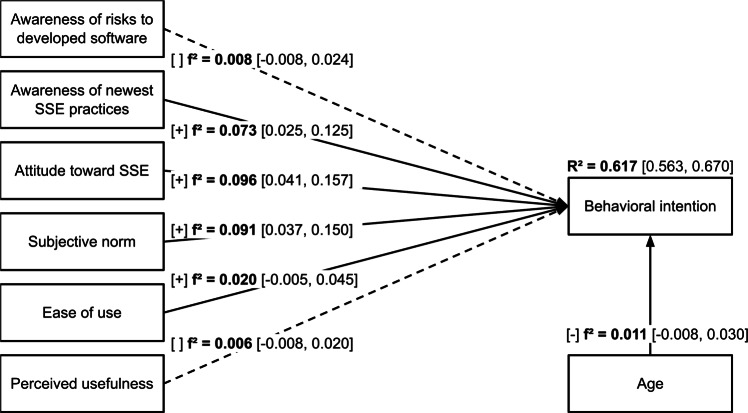
Structural model with effect sizes. $f^2 \ge 0.02$ – small effect, $f^2 \ge 0.15$ – medium effect, $f^2 \ge 0.35$ – large effect.

Effect sizes for the structural model are presented in Fig 1. The predictors explain a meaningful share of variance of *behavioral intention* ($R^2=0.617$, $95\%$ CI [0.563, 0.670]). Significant positive associations between *behavioral intention* and *awareness of newest SSE practices* ($beta=0.237$, $95\%$ CI [0.139, 0.334], $p<0.001$), *attitude toward SSE* ($beta=0.306$, $95\%$ CI [0.171, 0.441], $p<0.001$), *subjective norm* ($beta=0.249$, $95\%$ CI [0.157, 0.341], $p<0.001$), and *ease of use* ($beta=0.111$, $95\%$ CI [0.022, 0.201], $p=0.015$) indicate support for hypotheses H2, H3, H4 and H5, respectively. All these associations had small effect sizes ($f^2 \ge 0.02$). The results do not show support for hypotheses H1 ($beta=0.066$, $95\%$ CI $[-0.030, 0.163]$, $p=0.178$) and H6 ($beta=0.068$, $95\%$ CI $[-0.059, 0.194]$, $p=0.295$). The association with control variable age was negative and significant ($beta=-0.064$, $95\%$ CI $[-0.127, -0.001]$, $p=0.047$) albeit with a negligible effect size ($f^2 <0.02$).

### Experience with SSE

This subsection presents the results for the two subsamples, namely, the *pre-adoption* and the *post-adoption* subsamples. First, we conducted independent samples *t* tests as presented in Table 7.

**Table 7:** Descriptives with independent samples *t* tests for comparing means between the *pre-adoption* and the *post-adoption* subsamples.

	Full sample			Pre-adoption			Post-adoption			Independent samples	
Construct	*M*	*SD*	*Median*	*M*	*SD*	*Median*	*M*	*SD*	*Median*	*t*	*p*
Awareness of risks to developed software	4.15	0.68	4	3.96	0.73	4	4.30	0.60	4.5	$-5.570$	$<0.001$
Awareness of newest SSE practices	3.22	1.58	3	2.28	1.19	2	3.94	1.46	4	$-13.524$	$<0.001$
Attitude toward SSE	5.76	1.00	6	5.58	1.03	5.67	5.91	0.96	6	$-3.564$	$<0.001$
Subjective norm	3.03	1.08	3	2.62	1.03	3	3.34	1.02	3.33	$-7.510$	$<0.001$
Ease of use	3.17	0.83	3	2.92	0.79	3	3.37	0.81	3.33	$-6.009$	$<0.001$
Perceived usefulness	4.25	0.65	4	4.23	0.67	4.25	4.27	0.63	4	$-0.648$	0.517
Behavioral intention	4.91	1.46	5	4.36	1.51	4.67	5.34	1.27	5.67	$-7.418$	$<0.001$

These test indicate that mean scores in the *post-adoption* subsample are higher than mean scores in the *pre-adoption* subsample for all constructs except *perceived usefulness*. For this construct, the difference was non-significant.

Second, the structural model was tested on the *pre-adoption* and the *post-adoption* subsamples. Model fits for both subsamples are presented in Tables 8 and 9. These results suggest that the structural model fits both subsamples well.

**Table 8:** Fit indices of the structural model for the *pre-adoption* subsample.

Measure	Threshold	Estimate	Interpretation
$\chi ^2$		166.222	
*df*		149	
$\chi ^2 / df$	$\le 5$	1.116	Excellent
CFI	$\ge 0.90$	0.993	Excellent
TLI	$\ge 0.90$	0.991	Excellent
RMSEA	$\le 0.08$	0.024	Excellent
SRMR	$\le 0.08$	0.044	Excellent

CFI – comparative fit index; TLI – Tucker-Lewis index; RMSEA – root mean square error of approximation; SRMR – standardized root mean square residual.

**Table 9:** Fit indices of the structural model for the *post-adoption* subsample.

Measure	Threshold	Estimate	Interpretation
$\chi ^2$		235.534	
*df*		149	
$\chi ^2 / df$	$\le 5$	1.581	Excellent
CFI	$\ge 0.90$	0.977	Excellent
TLI	$\ge 0.90$	0.970	Excellent
RMSEA	$\le 0.08$	0.047	Excellent
SRMR	$\le 0.08$	0.042	Excellent

CFI – comparative fit index; TLI – Tucker-Lewis index; RMSEA – root mean square error of approximation; SRMR – standardized root mean square residual.

**Fig. 2:**
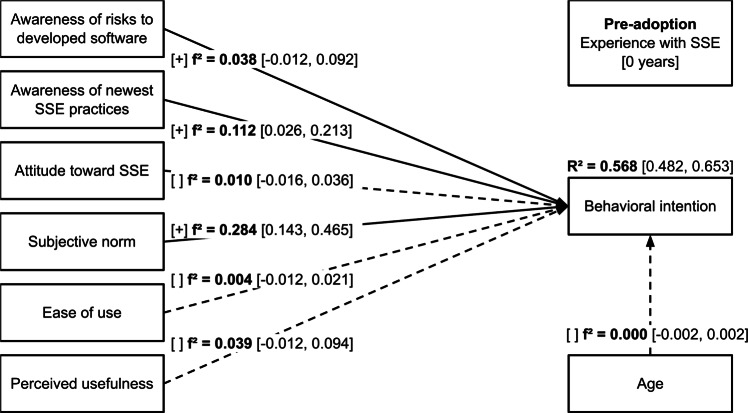
Structural model with effect sizes for the *pre-adoption* subsample. $f^2 \ge 0.02$ – small effect, $f^2 \ge 0.15$ – medium effect, $f^2 \ge 0.35$ – large effect.

**Fig. 3:**
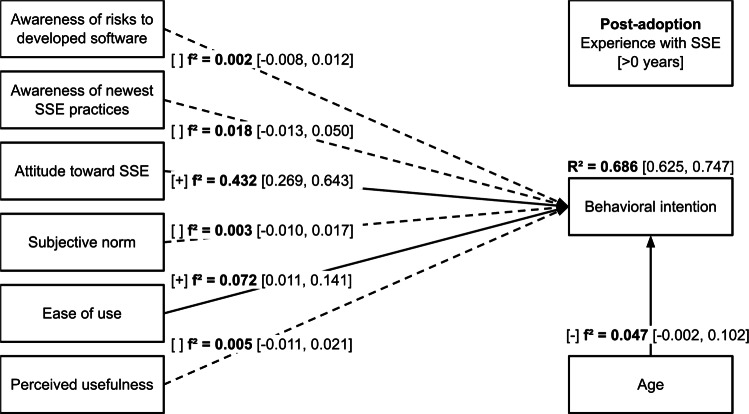
Structural model with effect sizes for the *post-adoption* subsample. $f^2 \ge 0.02$ – small effect, $f^2 \ge 0.15$ – medium effect, $f^2 \ge 0.35$ – large effect.

Effect sizes for the *pre-adoption* and *post-adoption* subsamples are presented in Figures 2 and 3, respectively.

The predictors explain a meaningful share of *behavioral intention* variance for both subsamples (*pre-adoption*: $R^2=0.568$, $95\%$ CI [0.482, 0.653]; post-adoption: $R^2=0.686$, $95\%$ CI [0.625, 0.747]). However, none of the associations were significant in both subsamples indicating a major shift in the determinants of behavioral intention from the pre-adoption to the post-adoption phase.

In the *pre-adoption* subsample, there were significant positive associations between *behavioral intention* and *awareness of risks to developed software* ($beta=0.139$, $95\%$ CI [0.001, 0.278], $p=0.049$), *awareness of newest SSE practices* ($beta=0.261$, $95\%$ CI [0.132, 0.391], $p<0.001$), and *subjective norm* ($beta=0.416$, $95\%$ CI [0.292, 0.541], $p<0.001$) which shows support for hypotheses H1, H2 and H4, respectively. The associations with *awareness of risks to developed software* and *awareness of newest SSE practices* had small effect sizes ($f^2 \ge 0.02$) while the association with *subjective norm* had a medium effect size ($f^2 \ge 0.15$). The association between *behavioral intention* and *perceived usefulness* was non-significant ($beta=0.170$, $95\%$ CI $[-0.018, 0.358]$, $p=0.076$) however had a small effect size ($f^2 \ge 0.02$) thus indicating some support for hypothesis H6. There was no support for hypotheses H3 ($beta=0.092$, $95\%$ CI $[-0.094, 0.277]$, $p=0.332$) or H5 ($beta=0.049$, $95\%$ CI $[-0.083, 0.181]$, $p=0.470$). The association with control variable age was non-significant ($beta=0.006$, $95\%$ CI $[-0.096, 0.107]$, $p=0.915$).

In the *post-adoption* subsample, there were significant positive associations between *behavioral intention* and *attitude toward SSE* ($beta=0.668$, $95\%$ CI [0.460, 0.875], $p<0.001$), and *ease of use* ($beta=0.193$, $95\%$ CI [0.077, 0.309], $p=0.001$) which shows support for hypotheses H3 and H5, respectively. The association with *attitude toward SSE* had a large effect size ($f^2 \ge 0.35$), and the association with *ease of use* had a small effect size ($f^2 \ge 0.02$). The results indicated no support for hypotheses H1 ($beta=-0.029$, $95\%$ CI $[-0.165, 0.106]$, $p=0.670$), H2 ($beta=0.107$, $95\%$ CI $[-0.028, 0.243]$, $p=0.121$), H4 ($beta=0.042$, $95\%$ CI $[-0.083, 0.167]$, $p=0.510$) or H6 ($beta=-0.059$, $95\%$ CI $[-0.251, 0.133]$, $p=0.549$). The association with control variable age was negative and significant ($beta=-0.122$, $95\%$ CI $[-0.202, -0.041]$, $p=0.003$), and had a small effect size ($f^2 \ge 0.02$).

### Summary

Table 10 provides a summary of hypothesis testing results.

**Table 10:** Summary of hypothesis testing results.

Hypothesis	Evidence	Conclusion
*H1*. Awareness of risks to developed software is positively associated with behavioral intention to practice SSE.	Significant positive association with small effect size in the *pre-adoption* subsample.	Partially supported
*H2*. Awareness of newest SSE practices is positively associated with behavioral intention to practice SSE.	Significant positive association with small effect size in the full sample and the *pre-adoption* subsample.	Partially supported
*H3*. Attitude toward SSE is positively associated with behavioral intention to practice SSE.	Significant positive association with small effect size in the full sample and large effect size in the *post-adoption* subsample.	Partially supported
*H4*. Subjective norm is positively associated with behavioral intention to practice SSE.	Significant positive association with small effect size in the full sample and the *pre-adoption* subsample.	Partially supported
*H5*. Ease of use is positively associated with behavioral intention to practice SSE.	Significant positive association with small effect size in the full sample and the *post-adoption* subsample.	Partially supported
*H6*. Perceived usefulness is positively associated with behavioral intention to practice SSE.	Non-significant association with a small effect size in the *pre-adoption* subsample.	Partially supported

## Discussion

This study is one of the first to study determinants of SSE practices adoption from varying theories and in different adoption phases. It makes a number of contributions to literature on adoption of SEE practices as well as adoption theories in general as presented in the following.

### Theoretical implications

This study has several theoretical implications. First, the results of this study indicate that both TPB and TAM explain adoption of SSE practices well albeit they may seem somewhat puzzling at first sight. Positive associations of behavioral intention with attitude toward SSE, subjective norm and ease of use indicate support for TPB. Even though the first two constructs are directly taken from TPB, *ease of use* (which is conceptually equivalent to effort expectancy^29^) is used as a replacement for the original TPB construct *perceived behavioral control* which is interpreted as perceived ease or difficulty of performing the behavior in several TPB studies, including in the SSE domain^11,62^. The associations with attitude toward SSE and subjective norm highlight the social and psychological aspects of adoption. A positive attitude toward SSE may originate from personal values or experiences that align with the principles of SSE. The role of subjective norm suggests that the social environment, including the opinions of colleagues and industry peers, plays a significant role in shaping behavioral intentions of individuals. This may be particularly relevant in environments where collaborative decision-making and peer influences are strong, such as in agile software development teams. These results are not fully consistent with^11^ where self-efficacy was not found to be associated with behavioral intention. This could be a consequence of including *ease of use* (a TAM construct) instead of *self-efficacy* (a decomposed TPB construct^62^) which may indicate a better fit of TAM for explaining this adoption determinant.

The overall support for TAM is however poorer than the support for TPB. Although subjective norm is associated with behavioral intention in the full sample, the third TAM construct, namely perceived usefulness, is not. On one hand, this appears to be consistent with the published TAM literature^14^. On the other hand, this is not quite consistent with the recent findings from the DOI literature highlighting the importance of relative advantage^13^ which is the DOI equivalent of perceived usefulness^18^. We may note that the association between relative advantage and adoption intention was barely significant in that study ($p=0.049$)^13^. Therefore, the support for this association is marginal at best in the literature. Interestingly, there is also some marginal support for this association for the pre-adoption subsample. Even though the association between perceived usefulness and behavioral intention is non-significant, it had a non-negligible (i.e., small) effect size. Perceived usefulness has been related to attitude toward behavior in the original TAM^24^ which may explain why this association is not significant^29^. These considerations suggest that there may be good reasons to consider adoption from a single theoretical perspective (i.e., either the TPB or the TAM perspective). However, there may be good reasons to consider adoption of SSE practices from an integrated TAM-TPB perspective, too, as explained by the next theoretical implication.

Second, this study advances the field by being among the first to investigate the intentions to practice SSE across different adoption phases. Specifically, the results of our study indicate a shift of significant determinants between the pre-adoption and the post-adoption phase. The interesting part is that neither TPB nor TAM explain adoption of SSE practices well on their own – but very well together, with the addition of new awareness constructs since $R^2$ ranged from 0.568 in the pre-adoption to 0.686 in the post-adoption phase.

In the *pre-adoption* subsample, only the association of behavioral intention with subjective norm was significant among TPB and TAM constructs while the association with perceived usefulness was non-significant with a small effect size. The medium effect of subjective norm in the pre-adoption phase highlights the influence of the social environment on initial adoption decisions. Developers might be more inclined to consider adopting SSE practices if they perceive it as a norm or expectation within their professional community (e.g., due to the opinions of peers, organizational culture or industry trends). Although subjective norm is a construct in both theories, these results indicate quite poor support for both TPB and TAM beyond subjective norm.

In the *post-adoption* subsample, there were significant associations of behavioral intention with attitude toward SSE (i.e., primarily a TPB construct) and ease of use (i.e., primarily a TAM construct). The association between behavioral intention and attitude toward SSE dominates in the post-adoption phase with a large effect size. This suggests that once developers adopt SSE practices their continued practicing may stem from a better understanding and appreciation of the value of SSE practices which develops through hands-on experience. Although having a small effect size, the association between behavioral intention and ease of use may indicate the importance of integrating SSE practices into existing workflows for continuing to practice SSE. Developers are more inclined toward continuing to use SEE practices that they find more straightforward and likely more compatible with their existing routines.

It is interesting to note that there was no overlap in significant determinants between the pre-adoption and post-adoption subsamples. Such differences presumably originate from an individual’s distinct perspectives on SSE practices during different phases of the adoption process. In the pre-adoption phase, individuals form their initial opinions based on the gathered information from their surroundings. Therefore, external factors, such as subjective norm and awareness, may be more meaningful. These factors should be considered in future studies focusing on the pre-adoption phase as well as studies on adoption of SSE practices in general. Other external factors may also play important roles thus future studies may seek to identify them. In the post-adoption phase, individuals adapt their opinions based on their own experience with SSE practices thus factors that are more closely related to them, such as ease of use and attitude toward SSE practices, gain prominence. These factors explain very well the continuance intention therefore future studies may consider them in their research models. This shift between the adoption phases can be therefore considered as a natural progression in the adoption life cycle. These findings also contribute to the broader adoption theory as there may be justifiable reasons to study different adoption phases separately contrary to the long-standing tendency to unify theories of adoption and (continued) use, such as UTAUT^29^ and its later variants. If adoption of SSE practices is nevertheless studied in a uniform way, relevant constructs from both adoption phases should be included in research models.

Third, this is among the first studies integrating awareness constructs in the research model. The addition of two awareness determinants (i.e., awareness of risks to developed software and awareness of newest SSE practices) provides a broader understanding of adoption of SSE practices. The positive association of behavioral intention with awareness of newest SSE practices indicates that staying updated with the latest advancements is a critical motivator for adopting SSE practices. This may be attributed to the frequently evolving nature of these practices since new threats and solutions emerge continuously. Similarly to TPB and TAM constructs, there were differences between the pre-adoption and post-adoption phase. Both awareness constructs were significant only in the pre-adoption subsample. This suggests that being aware of the potential risks to developed software and being aware of the solution to tackle them are among the key determinants of adoption of SSE practices. However, these factors appear to loose all importance after actually having experience with SSE practices.

### Practical implications

The empirical findings of this study also provide some implications for implementing DevSecOps in practice. First, the distinction between influencing factors in different phases of SSE adoption carries significant implications for software development managers. Our study shows that strategies to improve SSE adoption should be tailored to the specific adoption phase of practitioners. Managers should segment their teams into pre-adoption and post-adoption groups and apply targeted interventions that reflect the motivational and cognitive needs of each group. This approach enables more efficient and meaningful promotion of secure software practices.

Second, in the pre-adoption phase, behavioral intention is significantly influenced by developers’ awareness of both the risks to developed software and the newest SSE practices. Managers can take a proactive role by organizing short onboarding sessions, security awareness campaigns, or knowledge-sharing meetups that focus on real-world security incidents (e.g., injection vulnerabilities, authentication failures) and mitigation strategies. They may also provide curated learning materials or internal security newsletters to keep developers up to date with emerging SSE techniques and tools.

Third, shaping subjective norm is also particularly important in the pre-adoption phase. Managers can emphasize the benefits and necessities of compliance with industry standards, protection against rising cybersecurity threats, and alignment with peer and industry expectations. In practice, this may involve integrating secure coding practices into performance expectations, recognizing individuals or teams who demonstrate exemplary security behavior, or having security champions act as internal role models to establish secure behavior as the norm.

Fourth, for practitioners in the post-adoption phase, the focus should shift toward improving user experience and reinforcing positive attitudes toward security practices. Our results show that attitude toward SSE and ease of use are the most influential predictors of continued engagement. Therefore, managers should prioritize developer-friendly security tools and ensure they are well-documented, intuitive, and minimally disruptive to workflows. Collecting feedback from developers on tool usability and involving them in tool selection or configuration can significantly improve adoption.

Fifth, aligning SSE practices with developers’ values and preferences helps cultivate a more favorable attitude. Managers can demonstrate how secure coding reduces long-term rework, improves software maintainability, or supports team efficiency. Cross-functional collaboration with security teams and embedding security into agile activities (e.g., threat modeling in sprint planning) can further improve relevance and perceived usefulness.

Sixth, managers should also highlight the personal benefits of adopting SSE practices. Developers may be more motivated when they see security as a pathway to professional growth. Offering internal certifications or recognizing contributions in promotion and performance reviews can reinforce the value of security knowledge for individual career advancement.

### Limitations and future work

This study has some limitations that the readers should note. First, our research model included a limited number of SSE practice adoption determinants. While this approach was necessary to maintain a manageable survey length and minimize respondent fatigue, other relevant determinants may have been excluded from the study. Future studies may thus explore other constructs related to adoption of SSE practices. Second, our study relied on self-reported data. Self-reporting is susceptible to several biases, such as the social desirability bias according to which respondents answer questions in a manner they perceive as more favorable by others. There were some attempts to provide alternative measurement instruments (e.g., self-efficacy^63^) in the literature. Future studies may therefore employ other research designs and approaches to measure constructs, including observational studies, to provide a more accurate understanding of behaviors and attitudes toward SSE practices. Third, even though the use of the Prolific platform for participant recruitment is widely used in research, it is not without limitations due to potential selection bias. Future studies could expand the sample by employing other channels to reach a broader and more diverse population of software developers. Such an approach would enhance the representativeness of the sample thereby increasing the ecological validity of the findings. Additionally, cross-validation of the results with different sample sources could provide a more robust understanding of the factors associated with the adoption of SSE practices. Such approach would also address the potential limitation of our sample in which only 1.3% of surveyed declared themselves security experts. However, such results need to be interpreted in the broader agile software development context, where each role in the agile team can be done by more than one person, and one team member can do many roles^55,56^. Thus the 1.3% self-declared security experts are most likely those who in their careers predominantly assume the security expert role, while the rest of the 56.4% of the surveyed respondents who declared their experience with secure software engineering probably encountered the role of the security expert in their careers, even if it is not their preferred or dominant career role. Fourth, our study does not provide any concrete insights into why there are differences between the *pre-adoption* and *post-adoption* phases. Future studies, likely involving qualitative research methods, would be necessary to fill in this gap and provide meaningful insights that would further help to first understand the underpinnings of the adoption process and then to develop strategies for improving adoption of SSE practices in the industry. Fifth, we employed a cross-sectional survey design. While such an approach is consistent with prior research on behavioral intentions, it does not allow for causal inferences or the examination of changes over time. Future research employing longitudinal or experimental designs would therefore be valuable to validate the findings and provide insights into the dynamics of SSE adoption.

## Data Availability

All data generated or analysed during this study are included in this published article (and its Supplementary Information files)
